# Cytotoxic and Antifungal Activities of 5-Hydroxyramulosin, a Compound Produced by an Endophytic Fungus Isolated from *Cinnamomum mollisimum*

**DOI:** 10.1155/2012/689310

**Published:** 2012-02-16

**Authors:** Carolina Santiago, Chris Fitchett, Murray H. G. Munro, Juriyati Jalil, Jacinta Santhanam

**Affiliations:** ^1^Biomedical Science Programme, School of Diagnostic & Applied Health Sciences, Faculty of Health Sciences, Universiti Kebangsaan Malaysia, Jalan Raja Muda Abdul Aziz, 50300 Kuala Lumpur, Malaysia; ^2^School of Pharmacy, The University of Nottingham Malaysia Campus, Jalan Broga, 43500 Semenyih, Selangor, Malaysia; ^3^Department of Chemistry, University of Canterbury, Private Bag 4800, Christchurch 8140, New Zealand; ^4^Faculty of Pharmacy, Universiti Kebangsaan Malaysia, Jalan Raja Muda Abdul Aziz, 50300 Kuala Lumpur, Malaysia

## Abstract

An endophytic fungus isolated from the plant *Cinnamomum mollissimum* was investigated for the bioactivity of its metabolites. The fungus, similar to a *Phoma* sp., was cultured in potato dextrose broth for two weeks, followed by extraction with ethyl acetate. The crude extract obtained was fractionated by high-performance liquid chromatography. Both crude extract and fractions were assayed for cytotoxicity against P388 murine leukemic cells and inhibition of bacterial and fungal pathogens. The bioactive extract fraction was purified further and characterized by nuclear magnetic resonance, mass spectral and X-ray crystallography analysis. A polyketide compound, 5-hydroxyramulosin, was identified as the constituent of the bioactive fungal extract fraction. This compound inhibited the fungal pathogen *Aspergillus niger* (IC_50_ 1.56 **μ**g/mL) and was cytotoxic against murine leukemia cells (IC_50_ 2.10 **μ**g/mL). 5-Hydroxyramulosin was the major compound produced by the endophytic fungus. This research suggests that fungal endophytes are a good source of bioactive metabolites which have potential applications in medicine.

## 1. Introduction

Endophytic fungi live within plant tissue without causing apparent injury or symptoms to the host plant [1]. Some of these endophytes provide protection to their host plant from tissue-invading pathogens [2]. They are known to produce secondary metabolites with pharmacological importance such as antibiotic, antiviral, antidiabetic, and antiinflammatory compounds [3–6]. In some cases, endophytes in plants have been found to produce similar compounds as host plant [7].

The genus *Cinnamomum *belongs to the family Lauraceae. There are 21 species of *Cinnamomum *in Peninsular Malaysia [8]. Plants of this genus such as *C. zelyanicum* and *C. cassia* have been extensively used as antiseptic agents [9–11] and to treat bronchitis, cold, and sinus in herbal therapy [12]. Products of the plant such as cinnamon powder have been reported to possess minor antibacterial and antifungal activities on the skin [13]. Essential oil extract of *C. mollissimum* leaf has been found to have broad antifungal activity [14]. Recent findings describe the anticancer property of a constituent from *C. kotoense* leaf. This explains why plants of these species are also used to treat warts and certain cancerous tumours [15].

 As plants of *Cinnamomum *species have medicinal value, endophytic fungi from *C. mollissimum *were isolated and screened for antimicrobial activity. One endophytic fungal isolate was then selected to further evaluate its biological activity and to identify the bioactive compound which gives rise to the activity.

## 2. Methods

### 2.1. Isolation of Endophytic Fungi

Endophytic fungi were isolated from the plant *C. mollissimum* which was sampled at Universiti Kebangsaan Malaysia Forest Reserve, Selangor, Malaysia. The plant specimen voucher number 955 was deposited in the herbarium of Universiti Kebangsaan Malaysia. Plant leaf and stem pieces were surface sterilized [16] and cultured on potato dextrose agar and water agar to isolate endophytic fungi.

### 2.2. Preliminary Screening for Antimicrobial Activity

All fungal isolates recovered from the plant were screened for antimicrobial activity by using a modified agar plate-based assay [17]. In this assay, the fungal isolate was grown on potato dextrose agar (PDA) at 27°C for 14 days. The target organisms were streaked radial to the original growth of the fungal isolate in the centre of the plate. The target organisms comprising of bacterial and fungal pathogens were *Staphylococcus aureus, Bacillus subtilis, Escherichia coli, Pseudomonas aeruginosa, Candida albicans, Aspergillus niger, Trichoderma viridae, Fusarium solani, *and *Aspergillus fumigatus*. Antimicrobial activity was determined as growth inhibition of the target organism.

### 2.3. Extraction of Fungal Metabolites

One fungal isolate, coded as CB 007 (WA), which showed antimicrobial activity was selected for further evaluation. The fungus was cultured in 200 mL of potato dextrose broth with shaking mode at 140 rotations per minute, for two weeks at 27°C. The culture broth and mycelia were separated by filtration where the broth was extracted with an equal amount of ethyl acetate overnight. The fresh solvent extract was concentrated with a rotary evaporator to obtain a crude extract.

### 2.4. Fractionation of Crude Extract

An aliquot of the crude extract (250 *μ*g) was analyzed by reverse phase C_18_ high-performance liquid chromatography (HPLC) using the following gradient solvent system: 2 minutes (min) at 10% acetonitrile (ACN)/miliQ water (H_2_O); a linear gradient to 75% ACN/H_2_O over 12 min; isocratic at 75% for another 10 min; a linear gradient for 2 min to 100% ACN, isocratic at 100% ACN for 4 min then returned to 10% ACN/H_2_O over 2 min and reequilibrated for 8 min; with a flow rate 1 mL/min. The cycle lasted for 40 min, and the eluant was collected from 2.92 min to 24.92 min into 96-well polystyrene microtitre plates. A total of 88 fractions were collected, each fraction containing 250 *μ*L of sample. HPLC was performed on a Dionex system equipped with an ISCO Foxy Jr. sample collector using a reversed phase analytical column (Phenomenex Prodigy C18, 4.6 × 250 mm, 5 *μ*m) with photodiode array and evaporative light scattering detection (Alltech). 

### 2.5. Cytotoxicity Assay

The cytotoxicity assay was carried out on HPLC fractions according to methods previously described [18]. A total of 88 fractions in a microtitre plate were collected from HPLC analysis. Preparation of a daughter plate was made by transferring 50 *μ*L of extract fraction from each well of the master plate into another microtitre plate. The solvent was evaporated from the daughter plate by using a centrifugal evaporator machine before analysis for cytotoxicity against P388 murine leukaemia cells. For the P388 cytotoxicity assay, the following medium was used: *β*-Methoxyethoxymethyl, fetal calf serum (10%), penicillin (266 *μ*g/mL), streptomycin (132 *μ*g/mL), L-glutamine (2 mM), NaHCO_3_ (2.2 g/L), 4-(2-hydroxyethyl)-1-piperazineethanesulfonic acid (7.4 mM). The plate was incubated at 36°C for 3 days. After that, 20 *μ*L of thiazolyl blue tetrazolium (MTT) solution (3.8 mg/mL in phosphate buffered saline) was added to every well and the plate was incubated for 4 hours at 36°C. The formazon product was dissolved in hydrochloric acid in isopropanol (170 *μ*L, 0.08 M). Cell viability was determined by measuring the absorbance of every well at 540 nm. The absorbance of cell free control and the analyte free cell control was taken as 0% and 100% growth reference, respectively.

### 2.6. Antimicrobial Assay

A microtiter plate with HPLC fractions was prepared in the same way as for the P388 cytotoxicity assay. After evaporation of solvent in the microtitre plate, each well was added with 5% v/v methanol in water (10 *μ*L), suspension of test organisms (40 *μ*L; 5 × 10^5^ colony forming unit (CFU)/mL for *B. subtilis* and 2.5 × 10^3^ CFU/mL for *A. niger *and* A. fumigatus*), and medium (50 *μ*L; RPMI-1640 for *A. niger, A. fumigates,* and Mueller Hinton broth for *B. subtilis*). After incubation (27°C; 48 hours for *A. niger, A. fumigatus* and 37°C; 24 hours for *B. subtilis*), an MTT solution (20 *μ*L; 5 mg/mL) was added to all wells and further incubated (27°C; 4 hours). Subsequent to incubation, the formazon product was dissolved in dimethyl sulfoxide (100 *μ*L). Cell viability percentage was determined by measuring the absorbance of every well at 540 nm and subtracting the absorbance of cell free control. The absorbance of cell free control (solvent solution + medium) and the analyte free cell control (solvent solution + test organisms in medium) was taken as 0% and 100% growth reference, respectively. Cell growth inhibition was calculated as 100%—cell viability %.

### 2.7. Isolation of Bioactive Compound

Following determination of cytotoxic and antimicrobial activities of HPLC fractions, one bioactive fraction was selected for compound isolation. The active compound in this fraction as determined by its UV profile was collected via HPLC method using the same system as for the fractionation of crude extract. The active compound was collected manually in vials using its retention time and UV profile as a reference. This process was repeated up to ten times to obtain sufficient amount of the compound for structure elucidation. The sample was concentrated by blowing nitrogen gas onto the sample placed on a heating block set at approximately 30°C.

### 2.8. Structure Elucidation of the Bioactive Compound

NMR and mass spectrometry experiments were carried out on the bioactive compound. The NMR experiments, hydrogen 1 NMR, correlation spectroscopy, total correlation spectroscopy, heteronuclear single quantum coherence, heteronuclear multiple bond correlation, were recorded on a Varian INOVA 500 spectrometer at 23°C, operating at 500 MHz at 23°C, using a Capillary probe. Meanwhile, high resolution electrospray lonisation mass spectra (HRESIMS) were obtained on a Micromass liquid chromatography time-of-flight mass spectrometer.

The bioactive compound formed crystals when solvent from the compound sample was evaporated overnight using nitrogen gas at 8 to 10°C. X-ray crystallography of these crystals was carried out with a Bruker APEXII CCD area detector using graphite monochromised Mo K*α* (*γ* = 0.71073 Å) radiation. Data reduction was performed using SAINT software, and structures were solved by direct methods using SHELXS-97.

### 2.9. Determination of IC_50_ Values of the Bioactive Compound

Determination of IC_50_ for antifungal activity of the bioactive compound was done according to the standard M38-A method (NCCLS) with the compound used as the antifungal agent (concentration range 0.04 *μ*g/mL to 10 *μ*g/mL) and amphotericin B as a positive control. Meanwhile, for determination of IC_50_ value for P388 activity, the same method as in P388 cytotoxicity assay was employed with the bioactive compound tested at concentrations ranging from 1.99 *μ*g/mL to 4.10 *μ*g/mL. Calculation of the IC_50_ values was based on linear regression of plotted data.

## 3. Results

### 3.1. Isolation and Preliminary Screening of Endophytic Fungi

A total of 23 endophytic fungal isolates were isolated from *C. mollissimum*; 18 isolates were recovered from the leaves and 5 isolates from the plant stem. All fungal isolates were screened for antimicrobial activity. Four of the endophytic fungal isolates inhibited growth of bacteria and/or fungi (Table 1). Of these, only one isolate, CB 007 (WA), inhibited the growth of both bacteria (*B. subtilis*) and fungi (*A. niger *and *A. fumigatus*); therefore, this isolate was selected for further experimentation. 

### 3.2. Biological Activity of CB 007 (WA)

Fractions of CB 007 (WA) crude fungal extract obtained via HPLC were evaluated for antimicrobial and cytotoxic activity. Of 88 fractions tested, three factions were cytotoxic to P388 cells, while 17 fractions inhibited growth of *Bacillus subtilis*, 8 fractions inhibited growth of *Aspergillus fumigates,* and 3 fractions inhibited growth of *Aspergillus niger*. Of these fractions, only one fraction (C7) exhibited antibacterial, antifungal, and cytotoxic activity (Table 2). The UV profile of this fraction indicated the presence of one major compound (result not shown). 

### 3.3. Identification of the Bioactive Compound

Mass spectrometry, NMR analysis, and crystal structure determination revealed the pure compound in fraction C7 as 5-hydroxyramulosin.

**Figure 1:**
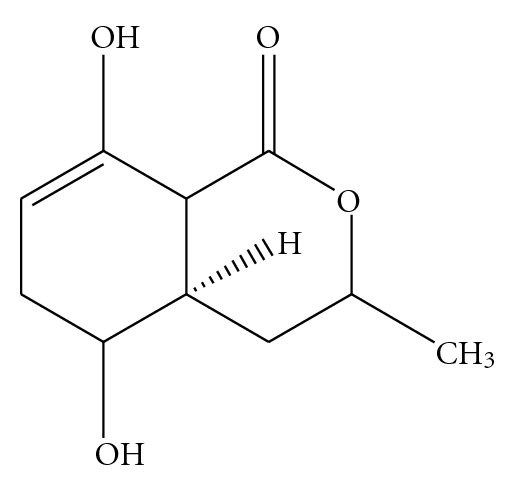
Chemical structure of 5-hydroxyramulosin C-carbon, H-hydrogen, O-oxygen.

5-Hydroxyramulosin (Figure 1): white crystals; UV (MeOH) *λ*_max⁡_ 202, 265, 301. HRESIMS *m*/*z* 199.1101 [M+H]^+^. Formula: C_10_H_14_NO_4_, *M* = 198.21. Crystals forming condition: 1 : 1 ACN : H_2_O at 8–10°C.

### 3.4. IC_50 _Values of the Bioactive Compound

The bioactive compound was also bioassayed to confirm its activity. The pure 5-hydroxyramulosin compound inhibited *A. niger* and was cytotoxic to P388 murine leukemic cells with IC_50_ values of 1.56 *μ*g/mL and 2.10 *μ*g/mL, respectively.

## 4. Discussion

*Cinnamomum* species is popularly used in herbal medicines due to its various therapeutic properties. Usually, essential oils from the leaf, inner bark, and stems of the plant have been used in herbal preparations [10]. On the other hand, endophytic microorganisms which occur in almost all plants are known to possess pharmacological properties. Therefore, the medicinal properties of a plant may be related to its endophytes. Endophytes from *Cinnamomum* species have been studied previously [19, 20]. However, there is no study to date on endophytes from *C. mollissimum.*

 In the present study, 23 fungal strains were isolated from *C. mollissimum, *of which 17.3% (4 isolates) demonstrated antimicrobial activity in preliminary screening. The agar-based screening assay employed is an easy method to identify active isolates for further experimentation and has been recommended as a rapid protocol in natural product research which enables determination of antimicrobial activity when a panel of pathogens are used [21]. One of the isolates, CB 007 (WA), which has not been identified yet but is morphologically similar to a *Phoma* sp., inhibited the bacteria *B. subtilis* and pathogenic fungi *A. fumigatus* and *A. niger*. As CB 007 (WA) was the isolate with the most broad-ranging activity, it was selected for further investigation to determine its bioactive component.

The approach taken to identify the active compound produced by the fungal isolate CB 007 (WA) was bioassay guided, where fractions of crude fungal extract were evaluated for biological activity prior to purification and structural elucidation of the compound in the active fraction. A microtiter-plate-based assay was employed in this study to test the HPLC fractions of crude fungal extract. This is an effective method to identify highly active fractions, subsequently reducing the time needed to isolate an active compound. The fractions were also evaluated for cytotoxicity against murine leukemic cells in order to select for fractions containing compounds with the most promising activity. As endophytes are known to produce a plethora of secondary metabolites [22], it is very likely that one organism is able to produce several active compounds. The most active fraction (C7) of the fungal extract displayed antibacterial, antifungal, and cytotoxic activity. This activity was consistent with the fungal isolate's antimicrobial activity in the initial agar plate based screening assay, except the fraction did not inhibit *A. fumigatus. *Nevertheless, other fractions of the crude extract were active against *A. fumigatus* (results not shown).

Purification and analysis of the active C7 fraction lead to the structural elucidation of 5-hydroxyramulosin which is a polyketide produced via pentaketide synthase action. Compounds grouped as polyketides are significant in natural product research because of their biosynthetic complexity and value in pharmaceutical industries. The polyketides comprise of toxins, antibiotics, therapeutic compounds, fungal melanins, and other pigments [23]. In fungi, pentaketide synthase is involved in the synthesis of metabolic precursors of melanin [24]. The culture CB 007 (WA) produces melanin when it grows mature (more than 14 days). The production of melanin in a fungus aids its survival especially under stressful conditions and increases its virulence potential [25, 26]. This may explain the biological activity observed in the fungal culture when it was initially screened in agar plate-based assay. Although 5-hydroxyramulosin is a product of pentaketide synthase, it is not known if this compound is involved in melanin synthesis.

 Fraction C7 which contained 5-hydroxyramulosin was initially observed to have inhibitory activity against *B. subtilis, A. niger,* and P388 murine leukemic cells. Nevertheless, when the pure compound 5-hydroxyramulosin was assayed against the same test subjects, inhibition was only detected for *A. niger* and P388 murine leukemic cells. The fraction may have contained a mixture of compounds, where the inhibitory effect against *B. subtilis* was due to the presence of other unidentified compound in the fraction. However, when 5-hydroxyramulosin was purified, it was ensured that only this compound was collected manually. 5-Hydroxyramulosin was previously identified in a marine-derived fungus *Phoma tropica,* and the compound was tested for inhibition of HIV-1 reverse transcriptase enzyme [27].

## 5. Conclusion

An endophytic fungus isolated from the terrestrial medicinal plant *Cinnamomum mollissimum* produced 5-hydroxyramulosin, a polyketide compound that had antifungal and cytotoxic activities. This compound may have pharmaceutical potential as it is highly active at low concentrations and is the major bioactive metabolite produced by the fungus.

## Figures and Tables

**Table 1:** Antimicrobial activity of endophytic fungi isolated from *C. mollissimum. *Target organisms were T01-*S. aureus*, T02-*B. subtilis*, T03*-E. coli*, T04-*P. aeruginosa*, T05-*C. albicans*, T06-*A. niger*, T07-*T. viridae*, T08-*F. Solani, *and T09-*A. fumigatus *(+: inhibition observed and −: no inhibition).

Endophytic fungal isolates	T01	T02	T03	T04	T05	T06	T07	T08	T09
CB 008 (WA)	−	−	−	−	−	−	−	−	−
CL 017 (WA)	−	−	−	−	−	−	−	−	−
CL 015 (WA)	−	−	−	−	−	−	−	−	−
CL 013 (WA)	−	−	−	−	−	−	−	−	−
CL 018 (WA)	−	−	−	−	−	−	−	−	−
CL 002	−	−	−	−	−	−	−	−	−
CL 005	−	−	−	−	−	−	−	−	−
CL 019 (WA)	−	−	−	−	−	−	−	−	−
CL 008	−	−	−	−	−	−	−	−	−
CL 012 (WA)	−	−	−	−	−	−	−	−	−
CL 014 (WA)	−	−	−	−	−	−	−	−	−
CL 003	−	−	−	−	−	−	−	−	−
CL 009	−	−	−	−	−	−	−	−	−
CB 002	−	−	−	−	−	−	−	−	−
CB 001	−	−	−	−	−	−	−	−	−
CL 006	−	−	−	−	−	−	−	−	−
CL 010	−	−	−	−	−	−	−	−	−
CB 006 (WA)	−	−	−	−	−	−	−	−	−
CL 012	−	−	−	−	−	−	−	−	−
CL 011	+	−	+	+	−	−	−	−	−
CL 007	+	−	+	+	−	−	−	−	−
CB 007 (WA)	−	+	−	−	−	+	−	−	+
CL 016 (WA)	−	−	−	−	−	+	−	−	−

**Table 2:** Biological activity of an HPLC fraction from CB 007 (WA) fungal crude extract. Results are expressed as % of target cells inhibited.

Assay	Fraction tested	Target cells	Cells inhibited (%)
Antibacterial activity	C7	*B. subtilis*	96.10
	Gentamycin (positive control)	*B. subtilis*	97.00
Antifungal activity	C7	*A. niger*	43.10
	Amphotericin b (positive control)	*A. niger*	96.6
Cytotoxicity	C7	P388 murine leukemia cells	88.60
